# AI-driven transformation in food manufacturing: a pathway to sustainable efficiency and quality assurance

**DOI:** 10.3389/fnut.2025.1553942

**Published:** 2025-03-13

**Authors:** Kushagra Agrawal, Polat Goktas, Maike Holtkemper, Christian Beecks, Navneet Kumar

**Affiliations:** ^1^School of Computer Engineering, KIIT Deemed to be University, Bhubaneswar, India; ^2^UCD School of Computer Science and CeADAR, University College Dublin, Belfield, Dublin, Ireland; ^3^Faculty of Mathematics and Computer Science, FernUniversität in Hagen, Hagen, Germany; ^4^ESM Division, ICAR - National Academy of Agricultural Research Management, Hyderabad, India

**Keywords:** artificial intelligence, circular economy, food manufacturing, predictive analytics, quality assurance, resource optimization, waste management

## Abstract

This study aims to explore the transformative role of Artificial Intelligence (AI) in food manufacturing by optimizing production, reducing waste, and enhancing sustainability. This review follows a literature review approach, synthesizing findings from peer-reviewed studies published between 2019 and 2024. A structured methodology was employed, including database searches and inclusion/exclusion criteria to assess AI applications in food manufacturing. By leveraging predictive analytics, real-time monitoring, and computer vision, AI streamlines workflows, minimizes environmental footprints, and ensures product consistency. The study examines AI-driven solutions for waste reduction through data-driven modeling and circular economy practices, aligning the industry with global sustainability goals. Additionally, it identifies key barriers to AI adoption—including infrastructure limitations, ethical concerns, and economic constraints—and proposes strategies for overcoming them. The findings highlight the necessity of cross-sector collaboration among industry stakeholders, policymakers, and technology developers to fully harness AI's potential in building a resilient and sustainable food manufacturing ecosystem.

## 1 Introduction

The escalating demands for sustainable solutions in food manufacturing emphasize the industry's critical role in addressing global environmental challenges, resource inefficiencies, and quality inconsistencies. Artificial intelligence (AI) is emerging as a transformative force, offering innovative pathways to *optimize production, minimize waste*, and *enhance sustainability*. However, despite rapid advancements, there remains a significant gap in understanding how AI applications can be effectively integrated into food manufacturing to meet sustainability goals. The integration of advanced technologies, including AI-driven tools, predictive analytics, and automation, has redefined traditional processes, emphasizing the inseparability of technological advancement and ecological sustainability (1). These developments align with the overarching concept of planetary health, which highlights the interconnection between human wellbeing and the Earth's ecosystems (2).

This study aims to fill this knowledge gap by systematically analyzing AI's role in transforming food manufacturing processes, focusing on efficiency improvements, environmental benefits, and real-world implementation challenges. Recent advancements in Industry 4.0 & 5.0 technologies, such as cyber-physical systems, Internet of Things (IoT) devices, and data-driven methodologies, have enabled significant improvements in the food supply chain (3). These innovations facilitate real-time monitoring and optimization of production processes, enhancing resource allocation, reducing waste, and improving product quality (4). Furthermore, AI-driven tools have proven instrumental in predictive maintenance and operational efficiency, transforming conventional systems into highly optimized and scalable frameworks (5, 6). Despite these technological breakthroughs, many food manufacturers face barriers to AI adoption, including a lack of clear implementation strategies, high infrastructure costs, and resistance to change.

The increasing consumer demand for environmentally friendly production methods and healthier food products has also driven a shift toward sustainability-focused manufacturing. Digitalization strategies, including AI-enabled analytics and smart sensors, offer practical solutions for reducing resource consumption and ensuring consistent product quality (7, 8). AI further supports the implementation of circular economy principles by enabling the repurposing of food by-products and improving demand forecasting to prevent overproduction (2, 9). However, several challenges hinder the widespread adoption of AI in food manufacturing. These include the difficulty of integrating AI into legacy systems, a shortage of skilled professionals, and ethical concerns related to data privacy and algorithmic bias (3, 10). Overcoming these barriers is imperative to unlocking the full potential of AI and ensuring its transformative impact extends across the entire food manufacturing ecosystem, from production to distribution and waste management.

To ensure a structured and comprehensive assessment of AI applications in food manufacturing, this study follows a literature review methodology. The review process involved a structured search across databases including Scopus, and Web of Science, applying predefined inclusion and exclusion criteria. Studies published between 2019 and 2024 were considered, focusing on peer-reviewed journal articles and conference proceedings. Gray literature and non-English studies were excluded to maintain research quality. Keywords such as “*Artificial Intelligence in Food Manufacturing," “AI for Sustainability in Food Processing,"* and “*AI-driven Quality Control in Food Industry"* were used to retrieve relevant articles. To enhance reliability, two independent reviewers conducted data extraction and categorized the findings.

Building on this foundation, this study provides a comprehensive review of AI-driven advancements in food manufacturing, addressing key industry needs such as:

- Optimizing efficiency: using AI tools like predictive analytics and machine learning (ML) to streamline workflows and reduce waste.
- Advancing sustainability: reducing environmental footprints and supporting circular economy practices.
- Addressing critical adoption barriers: including infrastructural challenges, ethical considerations, and economic constraints, with innovative strategies to ensure seamless integration into legacy systems.
- Proposing forward-looking solutions: aligning AI advancements with global sustainability goals, fostering resilience and scalability within the food manufacturing ecosystem.
- Bridging technological innovation with ecological responsibility: offering actionable frameworks for stakeholders to navigate opportunities and challenges.

By systematically analyzing existing literature and industry trends, this study bridges the gap between AI's potential and its practical implementation in food manufacturing. The insights presented in this review could serve as a valuable resource for industry stakeholders, policymakers, and researchers seeking to leverage AI for sustainable, efficient, and high-quality food production.

## 2 Methods

### 2.1 Search strategy and eligibility criteria

This literature review adhered to the Preferred Reporting Items for Systematic Reviews and Meta-Analyses (PRISMA) guideline (11). A comprehensive search was conducted across Scopus, and Web of Science to identify relevant peer-reviewed studies published between 2019 and 2024. The search terms were designed using a combination of controlled vocabulary and free-text keywords related to *AI applications in food manufacturing, sustainability, and quality assurance*. The search strings included:

- “Artificial Intelligence" OR “AI-driven technologies" AND “Food Manufacturing" OR “Food Processing".
- “Machine Learning" OR “Deep Learning" AND “Sustainable Food Systems".
- “Predictive Analytics" AND “AI for Food Waste Management".

Searches were concluded in January 2025, ensuring the inclusion of the most recent advancements in AI-driven food manufacturing innovations.

The inclusion criteria were:

- Peer-reviewed primary research articles, systematic reviews, and meta-analyses focused on AI applications in food manufacturing, sustainability, and quality assurance.
- Studies that explicitly discuss the implementation of AI in food production, waste management, or sustainability efforts.
- Research published in English-language journals within the time-frame 2019–2024 to ensure relevance to current technological trends.
- Studies providing empirical insights into *AI-driven predictive modeling, resource optimization, quality control, and circular economy practices* in food manufacturing.

The exclusion criteria were:

- Protocol papers, gray literature (*e.g*., dissertations, white papers, and technical reports), editorials, and opinion pieces.
- Non-English publications due to language constraints in this review.
- Studies that lacked substantial discussion of AI applications in food manufacturing or focused on unrelated industries.
- Publications prior to 2019, as AI's role in food manufacturing has significantly evolved over the past decade, making older studies less relevant.

### 2.2 Data screening, extraction, and synthesis

The selection process followed a rigorous two-stage screening process to ensure the relevance and quality of the included studies. Initially, titles and abstracts were screened using COVIDENCE program (12) and a custom Python algorithm developed by the authors to filter studies based on predefined inclusion and exclusion criteria. Studies that passed the preliminary screening underwent a full-text review, where two independent reviewers (KA and PG) assessed the articles based on relevance, quality, and methodological rigor. Any discrepancies in selection were resolved through consensus or consultation with a third reviewer. The review methodology followed a narrative synthesis approach, summarizing key themes across selected studies, identifying emerging trends, technological gaps, and challenges in AI-driven food manufacturing.

### 2.3 Data items and variables of interest

Data extraction was performed using a structured data extraction form developed in Microsoft Excel and a custom Python algorithm developed by the authors for qualitative synthesis. The key variables collected included:

- AI implementation details: type of AI technology used (*e.g*., machine learning, deep learning, predictive analytics).
- Sustainability impact: resource optimization, waste reduction, energy efficiency, and circular economy applications.
- Quality control measures: AI-driven food safety solutions, real-time monitoring, and process automation.
- Challenges and adoption barriers: ethical concerns, regulatory challenges, infrastructure limitations, and economic constraints.
- Key findings and recommendations: summary of results, proposed AI-driven strategies, and future research directions.

All extracted data were independently verified by two reviewers, ensuring accuracy and consistency in analysis. Any disagreements were resolved through discussion to maintain methodological integrity. The synthesized findings from the reviewed studies are presented in the following sections, detailing AI's transformative impact on food manufacturing.

## 3 Key challenges in traditional food manufacturing

Food manufacturing involves the large-scale production, processing, and packaging of food products for distribution and sale (13–15). It transforms raw agricultural materials, such as grains, fruits, vegetables, meat, and dairy, into finished goods that are safe, convenient, and appealing to consumers. This process includes sourcing raw materials from farms, fisheries, or other agricultural enterprises, followed by processing through mechanical techniques (*e.g*., cutting, grinding, mixing), thermal treatments (*e.g*., cooking, pasteurizing, sterilizing), and chemical methods to enhance shelf life and quality (16–20).

Processed food products are packaged in containers such as cans, boxes, bottles, or pouches to maintain freshness and enable efficient storage and transportation. Quality control is of crucial importance for food manufacturing (21), with stringent checks conducted to meet regulatory standards, such as those set by the Food and Drug Administration (FDA)^1^ in the United States or the Food Safety and Standards Authority of India (FSSAI).^2^ These measures ensure that the final products are safe for consumption and free of contaminants (22, 23).

The industry encompasses diverse products, including processed foods, ready-to-eat meals, beverages, and functional foods enriched with nutrients. However, traditional food manufacturing faces significant challenges:

- Resource-intensive processes: conventional food manufacturing heavily relies on energy, water, and raw materials. Processes like cooking, freezing, and sterilization consume vast amounts of energy (24), while cleaning and preparation require substantial water usage (25).
- High waste generation: by-products such as peels and residues are often discarded rather than repurposed, contributing to inefficiencies and environmental harm (26).
- Supply chain inefficiencies: poor coordination and logistical gaps lead to significant food losses, with ~14% of food globally lost between harvest and retail (27).
- Limited traceability: traditional systems often lack the ability to track products effectively through the supply chain, impacting food safety and recall efficiency (28).
- Overprocessing and overpackaging: excessive processing and packaging waste resources, degrade product quality, and increase environmental footprints (29).
- Environmental impact: energy consumption and waste decomposition contribute to greenhouse gas emissions (30), while water-intensive processes exacerbate water stress in vulnerable regions.

Table 1 summarizes these critical challenges, linking them to their respective environmental, operational, and societal impacts, along with key references supporting each aspect. Addressing these challenges requires a paradigm shift in traditional food manufacturing practices. Technological advancements, such as IoT, AI, and robotics, are pivotal in overcoming these inefficiencies by optimizing resource utilization, improving traceability, and reducing waste (37, 41). Integrating sustainable practices, such as repurposing by-products into bioenergy or animal feed (42), and adopting energy-efficient technologies can significantly mitigate environmental impacts while enhancing productivity and reliability.

**Table 1:** Key challenges in traditional food manufacturing and their impact.

**Challenge**	**Description**	**References**
Resource-intensive processes	Energy-demanding operations (*e.g*., cooking, freezing) and high water use for cleaning/preparation drive costs and environmental impact	(24, 25)
High Waste Generation	Discarded by-products (e.g., peels, residues) lead to methane emissions and inefficient resource utilization	(26, 31)
Supply chain inefficiencies	Poor logistics and coordination cause significant food losses, affecting resource efficiency and food security	(27, 32)
Environmental footprint	High greenhouse gas emissions from energy use and water stress in regions with limited freshwater exacerbate climate impacts	(33, 34)
Limited traceability	Inefficient tracking systems hinder food safety, recalls, and supply chain efficiency	(28)
Overprocessing and overpackaging	Excess processing degrades nutrition, while overpackaging increases waste and resource use	(29, 30)
Maintaining product quality	Variability in raw materials and process inefficiencies affect consistency, leading to waste and economic losses	(35, 36)
Adopting advanced technologies	High costs, skill gaps, and regulatory barriers slow IoT, AI, and robotics integration	(37, 38)
Unsustainable waste management	Limited adoption of circular economy practices (*e.g*., bioenergy conversion) and reliance on linear models exacerbate inefficiencies	(39, 40)

### 3.1 Persistent inefficiencies and resource-intensive processes

Food manufacturing is a critical component of the global food supply chain (43), yet it faces persistent inefficiencies and remains highly resource-intensive, creating challenges for sustainability, economic viability, and environmental health (44). The industry relies heavily on energy, water, and raw materials (45, 46). Thermal processes, such as cooking, drying, and freezing, are particularly energy-intensive, with refrigerated warehouses alone accounting for up to 20% of the industry's energy consumption (24). Similarly, water usage is significant (25), required for washing, cleaning, and preparing raw materials. The water footprint of processed foods often exceeds that of raw counterparts. Additionally, food processing generates considerable waste (26), including by-products such as peels and residues, much of which is underutilized or discarded.

Supply chain inefficiencies and logistical challenges exacerbate these issues (32). Poor coordination leads to significant food losses, with the FAO estimating that 14% of food is lost globally between harvest and retail (27). Equipment failures, unoptimized workflows, and production downtime further hinder operational efficiency, increasing costs and resource wastage. Over-processing and over-packaging degrade product quality, consume excess resources, and contribute to higher energy usage, with over-processing often diminishing the nutritional profile of food products (29). These inefficiencies collectively impact environmental sustainability. Energy consumption and waste decomposition contribute to greenhouse gas emissions (30), intensifying climate change. Water-intensive processes exacerbate water stress, particularly in regions where freshwater resources are already scarce. Additionally, the underutilization of food by-products adds to landfill waste, further straining environmental systems.

### 3.2 Environmental footprint and unsustainable waste generation

The food manufacturing sector exerts a profound influence on environmental sustainability due to its intensive energy use, significant water consumption, and extensive waste generation. As a pivotal element of global food production, the sector's ecological footprint has come under increasing scrutiny (34). Energy-intensive processes, such as cooking, sterilization, and freezing, are primary contributors to greenhouse gas emissions, with refrigerated storage accounting for a substantial portion of energy consumption, further intensifying the industry's carbon footprint (33). Similarly, water-intensive operations, including washing raw materials and cleaning equipment, exacerbate water stress, particularly in regions already facing freshwater scarcity (47).

The challenge of waste generation compounds these issues, as substantial by-products, including peels, husks, and residues, are often discarded or underutilized, leading to increased methane emissions from landfill decomposition (31). Alarmingly, up to 40% of global food waste occurs during the manufacturing stage, representing a significant loss of both resources and economic value (48). The environmental burden is further heightened by effluents containing chemical additives, fertilizers, and preservatives, which pollute water systems and strain ecosystems (39). Additionally, the reliance on nonrenewable resources and linear production models undermines sustainability efforts, with limited adoption of circular economy practices, such as converting food waste into bioenergy or animal feed (40).

To address these pressing environmental challenges, systemic transformations in food manufacturing are imperative. Enhancing resource efficiency, reducing waste, and adopting innovative waste management solutions are essential for mitigating the industry's environmental impact. Advancements in waste valorization technologies and the integration of renewable energy systems offer significant potential to align the sector with global climate objectives and the United Nations Sustainable Development Goals (SDGs), fostering a more sustainable and resilient food manufacturing ecosystem (39, 49).

### 3.3 Limitations in maintaining consistent product quality

Food manufacturing is a cornerstone of the global food supply chain, yet maintaining consistent product qualityposes significant challenges due to the inherent complexity of processes and variability in raw materials (35, 36). Product quality in this industry is affected by numerous factors, including inconsistent moisture content, temperature fluctuations during processing, and deviations in raw material properties (50). These variations can lead to production inefficiencies, diminished product quality, and increased waste, ultimately impacting consumer satisfaction and market competitiveness (51). For instance, in food extrusion processes, undetected abnormalities in feed materials, such as varying moisture content, can compromise the texture and quality of final products (50).

Moreover, ensuring quality standards across diverse product lines remains a daunting task. Traditional quality control measures, including manual inspections and routine sampling, are often inadequate in detecting subtle deviations, especially in high-speed production environments (52). While advanced technologies, such as near-infrared spectroscopy and process modeling, have demonstrated potential in addressing these limitations, their implementation requires significant investments and skilled operators (36). Inconsistent quality not only increases economic losses due to rework and recalls but also poses risks to brand reputation and regulatory compliance. Addressing these challenges demands a strategic integration of real-time monitoring tools, predictive quality control systems, and continuous process optimization to ensure consistency and uphold consumer trust in the evolving landscape of food manufacturing (38, 53).

## 4 AI-driven applications in sustainable food manufacturing

**Figure 1:**
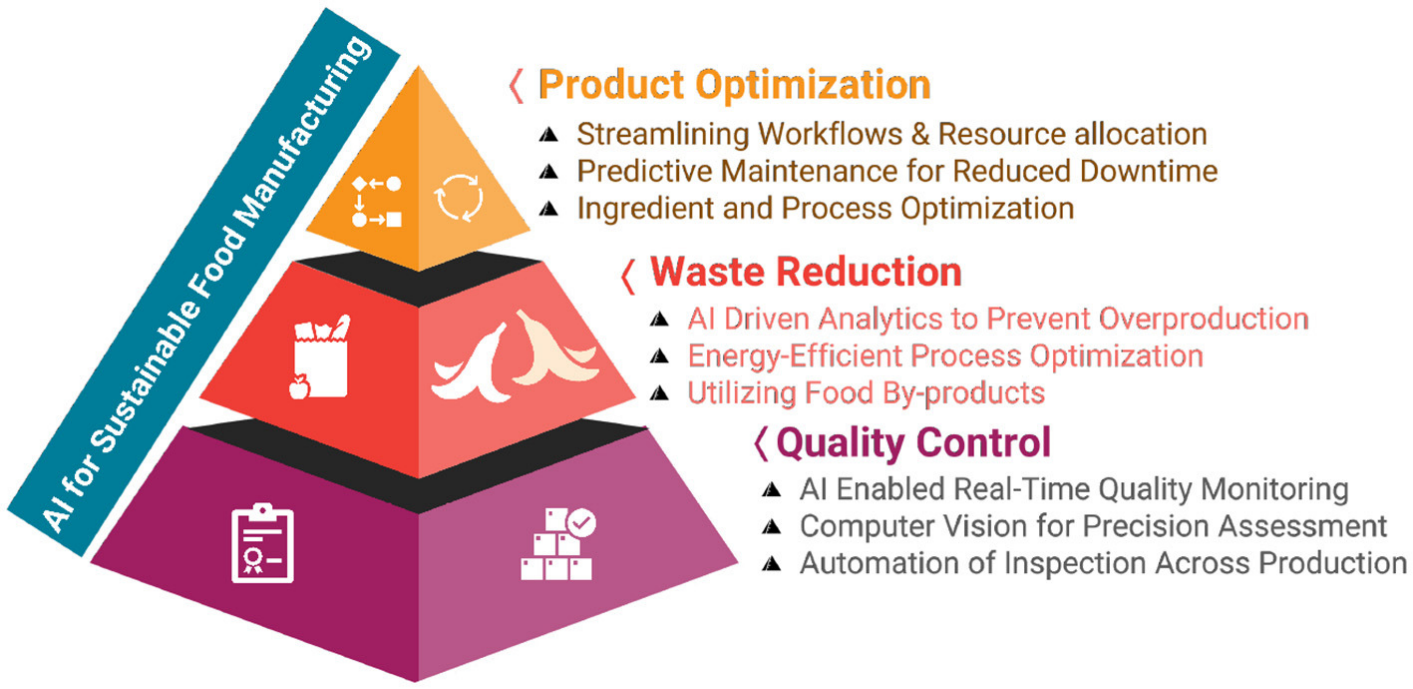
Illustration of AI-driven innovations enhancing sustainability in food manufacturing processes.

The challenges faced by traditional food manufacturing, such as resource-intensive processes, waste generation, and inconsistent product quality, demand innovative solutions to achieve sustainability goals. AI has emerged as a transformative tool to address these issues by optimizing processes, enhancing quality, and reducing environmental impacts. AI-powered heat drying technologies, for example, improve energy efficiency and reduce carbon footprints while preserving the nutritional and sensory properties of food products (54). Similarly, real-time AI monitoring in supply chain management minimizes food loss, ensures efficient resource utilization, and improves traceability (7, 55). Additionally, AI-based additive manufacturing enables personalized food production while maintaining high quality and safety standards through advanced data analysis (56). Figure 1 provides a visual representation of the diverse applications of AI in sustainable food manufacturing, highlighting its potential to streamline production, enhance efficiency, and foster innovation. Moreover, a detailed summary of key AI-driven applications and their associated benefits is provided in Table 2.

**Table 2:** AI-driven applications in sustainable food manufacturing and their benefits.

**AI application**	**Description and impact**	**Key benefits**
Heat drying optimization	Enhances energy efficiency while maintaining food nutritional and sensory qualities (54)	Reduced carbon footprint, preserved food quality, lower operational costs
Real-time supply chain monitoring	Tracks and optimizes supply chain operations, reducing waste and improving traceability (7, 55)	Reduced food loss, enhanced traceability, improved decision-making
Additive manufacturing	Enables personalized food production while maintaining quality and safety through data analysis (56)	Customizable food products, consistent quality assurance, reduced waste in production
Predictive maintenance	Anticipates equipment failures, reducing downtime and ensuring efficiency (57, 58)	Reduced unplanned interruptions, prolonged equipment lifespan, lower maintenance costs
By-product utilization	Repurposes food by-products into functional ingredients, promoting circular economy principles (59, 60)	Reduced waste generation, development of value-added products, enhanced sustainability
Real-time quality monitoring	Assesses food quality continuously using advanced sensors and AI (61, 62)	Improved food safety, minimized spoilage, enhanced consumer trust
Computer vision for quality assessment	Automates inspections with image processing and ML for accurate quality control (63, 64)	Enhanced defect detection, reduced manual errors, faster processing times
Inspection automation	Uses AI and ML to automate quality grading, ensuring consistency (65, 66)	Consistent quality assurance, reduced reliance on manual inspections, increased operational efficiency

Despite its transformative potential, AI integration faces barriers, including ethical concerns, data privacy issues, and the need for robust datasets (7, 55). However, the adoption of AI technologies offers significant opportunities to mitigate environmental impacts, optimize production, and align food manufacturing with global sustainability objectives. By addressing inefficiencies and environmental challenges, AI not only reshapes the industry but also sets a foundation for more resilient and eco-conscious food systems.

### 4.1 Product optimization

Product optimization is vital for sustainable food manufacturing, focusing on maximizing efficiency while minimizing resource usage. AI has transformed this domain, enabling precise control and adaptability in production workflows. By leveraging advanced data analytics algorithms, manufacturers can forecast production demands, automate scheduling, and optimize resource allocation, reducing errors and environmental impact. Advanced tools like Digital Twin (DT) systems allow manufacturers to simulate processes, benchmark sustainability metrics, and refine operations for greater efficiency (67, 68). AI-driven process optimization enhances shelf-life extension, particularly in dairy production, where it predicts spoilage risk and optimizes storage conditions (69). Additionally, AI-driven resource allocation models inspired by frameworks such as Unified Modeling Language (UML) enhance production adaptability (70, 71).

#### 4.1.1 Streamlining workflows and resource allocation

Streamlining workflows and optimizing resource allocation are critical to improving sustainability in food manufacturing. Lean manufacturing strategies, particularly in regions like Brazil and Peru, have reduced inefficiencies and waste, achieving annual research growth of 5.96% since 2019 (67). Technologies such as IoT and DT systems provide real-time tracking and virtual simulations to optimize production processes and benchmark sustainability metrics (68). AI enhances these efforts by enabling predictive analytics and automating resource allocation. Models inspired by biological systems, like endocrine regulation principles, and frameworks such as UML align dynamic production needs with available resources (70, 71). These innovations reduce bottlenecks, improve scheduling, and minimize idle time, contributing to cost savings and environmental sustainability.

#### 4.1.2 Predictive maintenance for reduced downtime

Predictive maintenance (PdM) uses advanced data analytics and ML to preempt equipment failures, ensuring smooth operations and reducing downtime. Techniques like Xtreme Gradient Boosting and ARIMA-ANN hybrid models enable early fault detection, while vibration sensors provide real-time monitoring to prevent interruptions (57, 58). Hybrid algorithms improve accuracy in identifying potential issues, which is critical in preventing spoilage and delays. By extending equipment lifespan and improving operational reliability, PdM reduces energy use and material waste, contributing to sustainability goals. Additionally, data mining offers insights into performance trends, helping manufacturers maintain consistent quality and reliability (72–74). This proactive approach ensures economic efficiency while minimizing environmental impact.

#### 4.1.3 Ingredient and process optimization for cost-efficiency

Ingredient and process optimization leverage tools like data envelopment analysis (DEA) and AI to enhance cost-efficiency without compromising quality. DEA evaluates multiple input-output relationships, facilitating cost-effective mixture designs (75). AI systems generate least-cost formulations based on real-time ingredient costs and inventory data, optimizing resource utilization and reducing expenses (76). Case studies highlight innovations such as incorporating vegetable proteins in chicken nuggets, which improve nutritional value and texture while reducing costs (77). Heat exchanger efficiency is significantly improved through AI-based modeling, which predicts and regulates thermal exchange in food manufacturing environments (78). These approaches demonstrate how targeted optimization can balance economic gains, product quality, and sustainability, fostering growth in the food manufacturing industry.

### 4.2 Waste reduction and circular economy

The food manufacturing industry plays a dual role as a critical economic driver and a significant contributor to waste and environmental challenges (79–81). Integrating AI into food manufacturing offers transformative solutions to reduce waste and align practices with circular economy principles (60, 82). Waste reduction focuses on minimizing excess production, optimizing resource utilization, and mitigating environmental harm. The circular economy further advances these efforts by promoting the reuse, recycling, and repurposing of materials to create a closed-loop system that maximizes resource efficiency (59, 83).

AI-driven technologies empower manufacturers to implement smarter production strategies, minimize inefficiencies, and repurpose by-products, reducing environmental footprints (80, 82). For example, predictive analytics and real-time monitoring enable precise inventory management and energy optimization (79, 81). By aligning production with market demand and utilizing by-products, manufacturers achieve cost savings and sustainability goals (59, 60). The following subsections explore specific AI-driven solutions in preventing overproduction, optimizing energy efficiency, and utilizing food by-products for secondary purposes.

#### 4.2.1 AI-driven analytics to prevent overproduction

Overproduction in food manufacturing leads to significant waste, primarily due to inaccurate demand forecasting and inefficient resource management. AI-driven analytics provide advanced solutions to align production volumes with actual market demands, thereby mitigating overproduction. AI systems can analyze extensive datasets, including historical sales, market trends, and external factors such as weather patterns, to predict consumer demand with high precision. This process, known as demand sensing, enables manufacturers to adjust production schedules proactively in response to real-time data, reducing the likelihood of overproducing perishable goods (84).

For instance, technologies like digital twins simulate production processes and optimize resource use in real-time, minimizing inefficiencies (80). Big data analytics further enhance demand forecasting by providing insights into consumer behavior and market trends. These capabilities enable just-in-time production strategies, reducing excess inventory and associated waste. The adoption of Industry 4.0 technologies, including AI, has also addressed barriers to circular economy practices in food supply chains (79). However, the effectiveness of AI-driven analytics depends on the quality and comprehensiveness of the data collected. Manufacturers must invest in robust data collection and management systems to ensure accurate demand forecasting. Moreover, integrating AI solutions requires addressing challenges such as high implementation costs, the need for skilled personnel, and the development of standardized governance frameworks to manage ethical considerations (85).

#### 4.2.2 Energy-efficient process optimization

Energy efficiency is pivotal for sustainable food manufacturing, addressing both environmental impacts and operational costs. Optimization techniques, such as those applied in agricultural systems, demonstrate potential for enhancing energy efficiency in food production (82). AI-powered heat drying technologies improve energy efficiency and reduce carbon footprints while preserving the nutritional and sensory properties of food products (86). Digitalization amplifies these efforts by enabling precise control and resource management, facilitating cost-effective production aligned with circular economy principles (81). AI and IoT technologies monitor energy usage across production lines, optimizing processes to minimize waste and reduce carbon emissions. These integrated strategies offer dual benefits: advancing sustainability goals and delivering economic advantages. To fully leverage these benefits, widespread adoption of energy-efficient practices remains critical.

#### 4.2.3 Utilizing food by-products for secondary purposes

Utilizing food by-products offers a transformative approach to sustainable food manufacturing, fully embracing circular economy principles. By reimagining by-products as resources rather than waste, manufacturers can reduce environmental impact and enhance product portfolios. Studies highlight the potential of by-products, such as dietary fibers, to serve as functional ingredients in various applications (59, 60). Innovative uses of by-products include incorporating fruit peels into health-oriented powders or enriching bakery items with dietary fibers. This not only minimizes waste but also caters to the rising demand for sustainable and health-conscious products. However, consumer acceptance is critical. Effective marketing and communication strategies are needed to ensure the success of value-added products derived from food waste (83). Challenges such as high initial technology costs and regulatory uncertainties remain barriers to widespread adoption. Collaborative efforts among industry stakeholders, researchers, and policymakers are vital for scaling up these practices. Developing cost-effective technologies, understanding consumer preferences, and establishing supportive regulatory frameworks can further promote the integration of by-products into mainstream production.

### 4.3 Quality control and assurance

Maintaining high standards of quality control and assurance in food manufacturing is essential not only for consumer safety but also for sustainability. AI technologies are increasingly being integrated into these processes to address challenges like contamination, spoilage, and over-processing. The application of ML algorithms and computer vision enables real-time monitoring, predictive analytics, and automation, which help identify defects and deviations from quality benchmarks at every step of production. By leveraging these advanced technologies, manufacturers can ensure that products meet regulatory standards and consumer expectations, while simultaneously reducing waste and enhancing efficiency. This proactive approach to quality assurance is a key component in achieving sustainable food manufacturing practices.

#### 4.3.1 AI-enabled real-time quality monitoring

Advancements in AI and smart sensing technologies have transformed real-time quality monitoring in the food supply chain. Integration of advanced sensors and biosensors in food packaging allows continuous assessment of quality parameters such as gas production, humidity, temperature, and microbial growth (61). These technologies provide immediate feedback on food quality, enabling timely interventions to prevent foodborne illnesses and ensure consumer safety. For example, portable handheld devices for food quality inspection are increasingly used across production stages, reflecting the trend toward automated monitoring systems (61). In a complementary innovation, Wang et al. (62) developed a fluorescent metal-organic framework (MOF) system for real-time visual monitoring of food freshness, specifically tested on raw fish samples. The system provides immediate and accurate feedback on quality, enhancing assurance processes in food manufacturing. The incorporation of smartphone-based platforms for inspection further underlines the growing accessibility and efficiency of quality monitoring solutions (62). These advancements play a crucial role in modern food manufacturing, ensuring food safety while reducing waste.

#### 4.3.2 Computer vision and sensor technology for precision assessment

The application of computer vision and sensor technologies in food manufacturing is gaining traction for its ability to enhance precision in quality assessment. Computer vision techniques, as highlighted by Jackman et al. (63), have significantly improved the accuracy of quality evaluations in fresh meats. By leveraging image processing, manufacturers can automate inspections, reducing reliance on subjective evaluations and improving defect detection efficiency. Deep learning (DL) algorithms are instrumental in advancing precision assessment techniques. For instance, Imani et al. (64) explored the use of layerwise imaging profiles in quality control, demonstrating how ML and advanced imaging techniques can yield more reliable assessments. These technologies enable real-time monitoring, allowing manufacturers to address potential quality issues early in the production cycle. Such innovations enhance the integrity and safety of food products while maintaining high standards of quality (64).

#### 4.3.3 Automation of inspection across production stages

Automation of inspection processes is essential for maintaining consistent quality standards in food manufacturing. Intelligent food packaging systems, as discussed by Dodero et al. (65), are a promising innovation for real-time quality monitoring during production and storage. These systems utilize responsive materials to provide continuous data on key quality parameters such as temperature, humidity, and gas composition, ensuring product freshness and safety while reducing waste. Additionally, ML-based systems offer transformative potential in automating inspections. Hemamalini et al. (66) proposed an approach using efficient image segmentation and ML techniques to enhance quality grading and assurance processes. These automated systems provide precise and rapid quality assessments, minimizing reliance on manual inspections and ensuring only high-quality products reach consumers. By integrating these technologies, food manufacturers can achieve consistent quality control, compliance with safety standards, and improved operational efficiency (66).

## 5 Challenges and limitations in AI adoption

The integration of artificial AI in the food industry holds significant promise; however, it is accompanied by several challenges and limitations that hinder its widespread adoption. Table 3 summarizes the key barriers, including technical and infrastructural constraints, ethical considerations, and financial viability issues, which are further detailed in the subsections below.

**Table 3:** Challenges and limitations in AI adoption in the food industry.

**Challenge**	**Description**	**References**
Technical and infrastructural barriers	High computational requirements, lack of advanced data processing capabilities, and limited access to reliable infrastructure (*e.g*., high-speed internet, cloud computing). Integration with legacy systems and absence of standardized frameworks further complicate adoption	(87–92)
Ethical dimensions of data privacy and security	Concerns over data governance, transparency, and trust. The absence of robust security protocols increases vulnerability to breaches, impacting supply chain efficiency and consumer wellbeing. Ethical principles emphasize inclusiveness, equity, and explainability in AI systems	(93–100)
Financial viability and resource constraints	High initial costs for infrastructure and training, coupled with ongoing maintenance expenses, restrict adoption, especially for SMEs. Data accessibility and affordability remain critical challenges in resource-constrained regions.	(101–103)

### 5.1 Technical and infrastructural barriers

The adoption of AI in food manufacturing is hindered by significant technical and infrastructural challenges, which limit its full integration and impact on the industry. One critical barrier lies in the need for advanced data processing capabilities and reliable infrastructure, such as high-speed internet and cloud computing, to support the seamless operation of AI systems (91). Many food manufacturers, particularly small and medium-sized enterprises (SMEs), lack access to these resources, which are essential for deploying AI-driven solutions in real-time production environments (88). Additionally, the high computational requirements for AI algorithms, such as neural networks and predictive models, pose further constraints on existing hardware and software systems (92).

Another key challenge is the integration of AI technologies with legacy systems in food manufacturing facilities. Most traditional production lines are not equipped to accommodate advanced technologies like IoT sensors or digital twins, which are crucial for real-time monitoring and optimization (89). Retrofitting these systems requires significant investment and technical expertise, making it economically unfeasible for many manufacturers (90). Furthermore, the lack of standardized frameworks for data collection and sharing exacerbates these issues, preventing seamless interoperability between AI-driven tools and existing operational systems (87).

Addressing these barriers requires a collaborative effort among stakeholders, including policymakers, technology providers, and industry leaders. Investment in affordable and scalable solutions, such as edge computing and distributed ledger technologies, can enable broader adoption of AI in food manufacturing (88, 104). Moreover, initiatives to build robust digital infrastructures and provide training in AI technologies can empower manufacturers to transition toward smarter and more sustainable production processes. The integration of digital twin models and IoT-enabled monitoring systems has further potential to optimize resource use and enhance efficiency in production (89, 90). Additionally, advanced sensing technologies like near-infrared spectroscopy (NIRS) offer green analytical solutions that can facilitate real-time decision-making and promote sustainability (87). These efforts are essential to overcoming infrastructural limitations and unlocking the transformative potential of AI in the food industry (92, 105).

### 5.2 Ethical dimensions of data privacy and security in AI systems

The integration of AI in food manufacturing introduces complex ethical challenges, particularly concerning data privacy and security. AI-driven systems increasingly collect and analyze large volumes of sensitive data, including proprietary business information and consumer-related data, to optimize supply chains, enhance food safety, and predict market trends. However, this reliance on data raises critical questions about its secure storage, accessibility, and ethical use. According to Jacobs et al. (98), transparency and trust in digital collaborations are essential for maintaining ethical practices in data management. The absence of clear governance frameworks for data privacy can undermine stakeholder confidence and lead to significant ethical and operational risks.

The WHO's guidance on the Ethics and Governance of Artificial Intelligence for Health (99) offers a robust framework for addressing ethical considerations in AI. It outlines six core ethical principles—*autonomy, human wellbeing, transparency and explainability, responsibility and accountability, inclusiveness and equity, & responsive and sustainable* systems. These principles emphasize the need for inclusive and equitable AI systems that protect user privacy while fostering innovation. In the context of food manufacturing, such principles provide a foundation for self-governance and equitable data practices, ensuring that AI solutions serve the interests of all stakeholders, including marginalized communities (95, 96).

A major concern in this domain is the lack of standardized data governance practices, which can expose the food sector to significant risks. Karanth et al. (97) emphasize that breaches in data privacy can compromise predictions related to food safety and supply chain efficiency, ultimately impacting both consumer wellbeing and business sustainability. AI platforms that predict contamination risks or assess vulnerabilities in the supply chain, as highlighted by Chavan et al. (94), rely on comprehensive data analytics. However, without robust security protocols, these systems may inadvertently expose sensitive data to unauthorized access or misuse, creating vulnerabilities across the industry.

To address these challenges, a proactive approach to ethical AI application is essential. Collaboration among technology providers, regulators, and industry leaders can help establish guidelines that prioritize data privacy and foster trust. Friedlander and Zoellner (100) suggest that designing transparent and accountable AI systems is critical for mitigating ethical risks. Furthermore, incorporating explainability into AI algorithms, as advocated by Goktas (93), enhances stakeholder confidence by ensuring that AI decisions align with ethical standards and societal values. By adopting robust data governance policies and investing in secure, transparent AI solutions, the food manufacturing sector can balance innovation with the protection of privacy, ensuring ethical progress in the digital era.

### 5.3 Financial viability and resource constraints in AI adoption

The financial viability and resource constraints of adopting AI technologies significantly influence their implementation in the food industry. The high initial investment required for infrastructure, training, and operational adaptation presents a substantial challenge, particularly for SMEs. As noted by Jäggi et al. (101), globalization has intensified the complexity of food markets, necessitating advanced technological solutions to enhance supply chain management, productivity, and sustainability. However, these advancements often demand resources that are beyond the reach of smaller stakeholders, creating economic barriers that limit equitable adoption. Beyond initial investments, the ongoing costs of maintaining and upgrading AI systems further complicate their feasibility. For instance, Tsakiridis et al. (102) illustrate that while IoT-enabled AI tools such as precision irrigation systems deliver significant efficiency gains and waste reduction, their high installation and maintenance costs remain a deterrent for widespread adoption. Addressing these barriers requires innovative solutions, including the development of low-cost sensors and explainable AI models, which can increase both affordability and trustworthiness, thereby enabling broader access to AI-driven systems in agriculture and food production.

Another critical challenge lies in the data infrastructure required for AI applications. As Qureshi (103) observes, data-driven AI solutions necessitate extensive and high-quality datasets, which are often inaccessible in under-resourced regions. This data divide risks marginalizing vulnerable populations and regions, exacerbating existing inequalities (95). Bridging this divide demands targeted policies that foster equitable access to digital resources and investments in inclusive data collection systems. Public-private partnerships can play a pivotal role in sharing the financial burden, promoting collaboration, and scaling AI solutions in a manner that benefits diverse stakeholders in the food industry.

## 6 Future perspectives and innovations

The food manufacturing sector is undergoing a paradigm shift, driven by the convergence of technological advancements and the imperative for sustainable practices. Emerging technologies such as green AI and blockchain are poised to transform production processes by enhancing efficiency, reducing waste, and ensuring superior food safety and quality. Additionally, the adoption of circular economy principles and renewable energy solutions is reshaping the industry's operational landscape, aligning it with global sustainability objectives. As detailed in Table 4, the integration of cutting-edge technologies such as sustainable AI, IoT, robotics, and blockchain in food manufacturing addresses critical challenges while unlocking significant environmental and economic benefits. The following sub-sections delve deeper into the key areas highlighted in the table, offering a detailed overview of their applications, benefits, and implications for the future of food manufacturing:

**Table 4:** Key innovations and future perspectives in food manufacturing.

**Technology**	**Key innovations**	**Future opportunities and advancements**
Sustainable AI	- Real-time monitoring and predictive analytics (28) - Precision farming for optimized resource utilization (106) - Enhanced waste management (107)	- Democratizing AI adoption across all scales (108) - Seamless integration with Industry 5.0 principles (109)
IoT	- Sensor-driven decision-making (106) - Predictive maintenance for reduced downtime (110)	- Improved scalability and interoperability (111) - Integration with blockchain for traceability (111)
Robotics and autonomous systems (RAS)	- Automated sorting and defect detection (112) - Collaborative robots for safer workplaces (110)	- Enhanced human-robot interaction (110) - Greater efficiency in material handling (110)
Digital twins (DT)	- Virtual replicas for real-time process simulation (113) - Resource management optimization (114)	- Integration with machine learning for predictive analytics (114) - Addressing dynamic demands in production (113)
AI-driven innovations	- Personalized nutrition through ML (115) - AI-based quality assurance systems (116)	- Improved regulatory compliance (116) - Tailored food production for individual needs (115)
Circular economy and renewable energy	- Utilization of surplus ingredients and by-products (117) - Adoption of renewable energy solutions (107)	- Increased alignment with sustainability goals (108) - Enhanced energy efficiency across supply chains (109)

### 6.1 Emerging AI technologies shaping food manufacturing

The integration of advanced technologies, such as sustainable AI, IoT, and Robotics and Autonomous Systems (RAS), is redefining the future of food manufacturing. These innovations are pivotal in addressing critical challenges like sustainability, food safety, and adaptability to evolving market demands while simultaneously driving operational efficiency and resilience. As highlighted by Moses and Anandharamakrishnan (114), these technologies are vital in transforming traditional practices into highly automated and optimized systems aligned with Industry 5.0 principles.

#### 6.1.1 AI and IoT: transforming decision-making in food manufacturing

The convergence of AI and IoT enables real-time monitoring and data-driven decision-making across the food manufacturing supply chain. According to Misra et al. (28) and Ding et al. (106), AI-powered precision farming utilizes sensor-driven data to optimize resource utilization, improve crop yields, and enhance production efficiency. IoT devices, coupled with AI algorithms, offer predictive maintenance capabilities, minimizing equipment downtime and reducing waste in food processing operations. In logistics and supply chain management, AI optimizes resource allocation and reduces carbon footprints through intelligent routing and scheduling, contributing to the overall sustainability of food manufacturing (110). Emerging research further highlights the role of blockchain integrated with AI and IoT in ensuring end-to-end traceability in the supply chain, enhancing transparency, and reducing fraud (111). This synergy is expected to address scalability and interoperability challenges, paving the way for a seamless and efficient supply chain network.

#### 6.1.2 RAS: driving productivity and precision

The advent of RAS further amplifies the impact of AI in food manufacturing. As noted by Kumar and Konar (112), robotic systems not only automate these tasks but also improve consistency and reduce labor costs. Integration with AI allows for precise control over production processes, ensuring standardized quality and efficiency. For instance, robotic sorting systems equipped with machine vision can detect and remove defective products with remarkable accuracy, maintaining high food safety standards. Autonomous vehicles in manufacturing facilities further streamline material handling, optimizing workflow efficiency and reducing operational costs (110). Additionally, collaborative robots, or “*cobots*," are increasingly being utilized to enhance human-robot interactions in production environments, fostering a safer and more efficient workplace.

#### 6.1.3 Digital twins: virtual optimization of processes

The application of DT technology represents a significant leap in the optimization of food manufacturing processes. By creating virtual replicas of physical processes, DT facilitates real-time simulation, monitoring, and optimization of production workflows. According to Grewal et al. (113), DT enhances resource management, minimizes waste, and optimizes energy utilization, aligning with sustainability objectives. The adaptability and scalability of DT make it a valuable tool for addressing the dynamic demands of the food industry. Future advancements in DT are expected to integrate ML models for predictive analytics, enabling manufacturers to anticipate potential bottlenecks and implement proactive measures. This approach contributes to greater resilience and sustainability in the food manufacturing ecosystem (114).

#### 6.1.4 Industry 5.0 in food manufacturing

Industry 5.0 represents the next phase in industrial evolution, building upon the digital transformation of Industry 4.0 by integrating human-centric, sustainable, and resilient manufacturing principles. Unlike Industry 4.0, which emphasized automation, cyber-physical systems, and AI-driven decision-making, Industry 5.0 focuses on collaborative intelligence, where humans and advanced technologies work together to optimize production processes, enhance sustainability, and improve overall resilience (110, 118).

Key principles of Industry 5.0 in food manufacturing should sinclude:

- Human-AI collaboration: unlike fully automated systems in Industry 4.0, Industry 5.0 emphasizes human-machine interaction, where AI assists human workers in making informed decisions while ensuring flexibility and adaptability in manufacturing (119).
- Sustainability-driven production: the shift toward circular economy practices is accelerated under Industry 5.0, where AI, IoT, and robotics are leveraged to minimize waste, repurpose by-products, and reduce energy consumption (120).
- Resilient and adaptive systems: industry 5.0 fosters resilience by enabling smart manufacturing systems that can quickly adapt to supply chain disruptions, ensuring a stable and sustainable food production process (121).

The integration of Industry 5.0 principles into food manufacturing offers significant advantages, such as enhancing AI-driven predictive maintenance, promoting sustainable production models, and supporting customized food manufacturing through robotics and personalized nutrition technologies. By leveraging these innovations, the food industry can transition toward a more efficient, environmentally responsible, and consumer-focused production ecosystem (110).

#### 6.1.5 Advancing AI innovations: personalized nutrition and enhanced quality assurance

Emerging AI technologies are driving innovations in personalized nutrition and quality assurance in food manufacturing. ML models enable the customization of food products to cater to individual nutritional needs and preferences, as emphasized by Viejo et al. (115). Additive manufacturing, powered by AI, facilitates the creation of tailored food items with enhanced nutritional profiles, bridging the gap between health and convenience (56). AI-based quality control systems are also advancing food safety protocols by leveraging computer vision and DL to detect contaminants, assess freshness, and monitor shelf life. These technologies significantly enhance the accuracy and efficiency of quality assurance processes, ensuring compliance with stringent regulatory standards (115, 116).

#### 6.1.6 Overcoming barriers and shaping the future path

Despite the transformative potential of AI technologies, several challenges hinder their widespread adoption in food manufacturing. High implementation costs, data dependency, and ethical concerns related to privacy and bias remain significant barriers (122). Addressing these challenges requires collaborative efforts among governments, academia, and industry stakeholders to develop cost-effective solutions and establish robust regulatory frameworks (87). Future research should focus on enhancing the interpretability and transparency of AI models to build trust among stakeholders. Moreover, initiatives to upskill the workforce and promote interdisciplinary collaboration are essential for maximizing the potential of these technologies. The road ahead involves leveraging AI to achieve a balance between innovation, sustainability, and ethical considerations in food manufacturing (110, 114).

### 6.2 Toward a greener future: AI in sustainable food production

**Figure 2:**
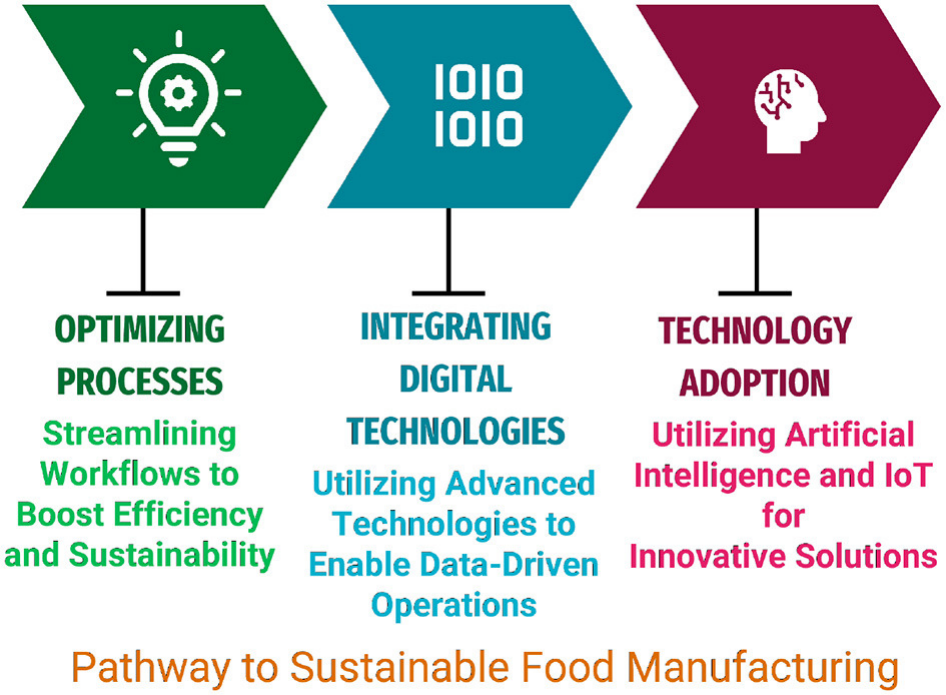
A framework illustrating the three pillars of sustainable food manufacturing: *optimizing processes, integrating digital technologies*, and *the adoption of AI and IoT technologies* for innovative and efficient production.

The integration of AI with big data and IoT is driving significant advancements in sustainability within food manufacturing. These technologies improve operational efficiency, optimize resource utilization, and reduce waste. Misra et al. (28) emphasize how real-time monitoring and predictive capabilities enable manufacturers to enhance workflows, minimize losses, and address environmental challenges, aligning with global sustainability targets. Koebe (108) underline the broader applicability of digital technologies, including AI, in advancing the United Nations' SDGs. Leveraging scalable AI-driven models and digital platforms supports sustainable practices and fosters global adaptability, even in emerging markets. One impactful innovation in food manufacturing is AI-driven 3D food printing, which reduces material waste while enabling precision production of customized food items tailored to individual preferences. By incorporating surplus ingredients and by-products into formulations, manufacturers actively promote circular economy principles, significantly reducing food waste (117). The broader framework for sustainable food manufacturing, as depicted in Figure 2, highlights the three essential pillars: *optimizing processes, integrating digital technologies*, and *adopting AI and IoT solutions*. This framework underlines the importance of combining technological innovation with process efficiency to achieve long-term sustainability goals.

AI-powered energy management systems further advance sustainability by optimizing energy consumption across the food supply chain, reducing carbon footprints, and lowering operational costs (28). Furthermore, the integration of AI into waste management systems fosters sustainable urban growth, as highlighted by Singagerda et al. (107), who emphasize AI's ability to influence public behavior, enhance resource efficiency, and align with SDGs 11, 12, and 13. Despite these advancements, challenges such as high implementation costs and the need for skilled personnel persist. However, the adoption of low-code AI platforms and Industrial IoT (IIoT) solutions, as demonstrated by Redchuk et al. (109), showcases a pathway for achieving energy efficiency and resource optimization while maintaining centrality of human expertise within the Industry 5.0 framework. Ultimately, AI's potential to foster resilience, reduce waste, and enhance customization paves the way for a sustainable, adaptive, and efficient food manufacturing ecosystem (28, 108, 117).

## 7 Conclusion

The integration of AI technologies into food manufacturing redefines the industry, enabling sustainable and efficient production systems. By addressing critical challenges such as resource optimization, waste reduction, and quality assurance, AI, together with IoT and robotics, transforms traditional processes into intelligent, interconnected systems capable of real-time monitoring and adaptive decision-making. This study contributes to the field by providing a structured analysis of AI applications in food manufacturing, offering practical insights for industry adoption, and identifying research gaps that need further exploration.

Findings from this review highlight that AI has the potential to:

- Enhance operational efficiency by automating workflows and optimizing resource use.
- Reduce food waste and environmental impact through AI-driven predictive analytics.
- Improve product quality control by integrating AI-based defect detection and real-time monitoring systems.
- Facilitate the transition to circular economy practices by repurposing food by-products.

Advanced sustainable AI applications are expected to seamlessly integrate into existing infrastructures, improving predictive maintenance, quality control, and operational efficiency. The adoption of AI-driven solutions accelerates the shift toward circular economy practices, optimizing the utilization of by-products and enabling precise resource allocation. However, realizing these benefits requires addressing existing challenges such as high implementation costs, ethical concerns, and the need for standardized AI governance frameworks. Thus, this study emphasizes the importance of multi-stakeholder collaboration among policymakers, technology developers, and food industry professionals to unlock AI's full potential in food manufacturing. By overcoming adoption barriers and leveraging AI-driven innovations, the industry can achieve a balance between technological advancement, environmental sustainability, and economic viability. The insights provided in this review contribute to ongoing discussions on the role of AI in shaping the future of sustainable food production, laying the groundwork for further research and policy development.

## Data Availability

The original contributions presented in the study are included in the article/supplementary material, further inquiries can be directed to the corresponding author.

## Footnotes

^1^ https://www.fda.gov

^2^ https://fssai.gov.in
